# A Study of the Influence of Measurement Volume, Blending Ratios and Sensor Precision on Real-Time Reconciliation of Grade Control Models

**DOI:** 10.1007/s11004-018-9740-3

**Published:** 2018-05-09

**Authors:** T. Wambeke, J. Benndorf

**Affiliations:** 1 Delft University of Technology, Stevinweg 1, 2628CN Delft, The Netherlands; 2 University of Technology Bergakademie Freiberg, Fuchsmühlenweg 9, 09599 Freiberg, Germany

**Keywords:** Grade control model, Reconciliation, Geostatistics, Ensemble Kalman filter, Data assimilation, Production data

## Abstract

The mining industry continuously struggles to keep produced tonnages and grades aligned with targets derived from model-based expectations. Deviations often result from the inability to characterise short-term production units accurately based on sparsely distributed exploration data. During operation, the characterisation of short-term production units can be significantly improved when deviations are monitored and integrated back into the underlying grade control model. A previous contribution introduced a novel simulation-based geostatistical approach to repeatedly update the grade control model based on online data from a production monitoring system. The added value of the presented algorithm results from its ability to handle inaccurate observations made on blended material streams originating from two or more extraction points. This contribution further extends previous work studying the relation between system control parameters and algorithm performance. A total of 125 experiments are conducted to quantify the effects of variations in measurement volume, blending ratio and sensor precision. Based on the outcome of the experiments, recommendations are formulated for optimal operation of the monitoring system, guaranteeing the best possible algorithm performance.

## Introduction

Traditionally, the mining industry has had mixed successes in achieving its production targets (Ward and McCarthy 1999; Vallee 2000; Tatman 2001; McCarthy 2003). The deviation of produced tonnages and grades from model-based expectations often results from a mismatch in scale between exploration data and short-term production targets (Benndorf 2013). For example, sparse data from holes drilled at relatively wide grids is by no means sufficient to characterise truck loads accurately, designated to be fed into the processing plant. Grade control (GC) drilling can reduce the uncertainty to some extent (Peattie and Dimitrakopoulos 2013; Dimitrakopoulos and Godoy 2014). The uncertainty can be further reduced by assimilating abundantly available sensor measurements into the GC model (Wambeke et al. 2018). Though abundantly available, sensor measurements are also significantly more noisy and do often characterise a blend of material originating from multiple extraction points. Recently, Wambeke and Benndorf (2017) developed an algorithm capable of handling these specific challenges.

Once new measurements become available, the algorithm updates GC model realisations $\mathbf {Z}_{t}(:, i)$ based on a weighted difference between a set of estimates $\mathcal {A}_t(\mathbf {Z}_{t-1}(:, i))$ and actual observations $\mathbf {d}_t$

1 $$\begin{aligned} \mathbf {Z}_t(:, i) = \mathbf {Z}_{t-1}(:, i) + \mathbf {W}_{t} (\mathbf {d}_t - \mathcal {A}_t(\mathbf {Z}_{t-1}(:, i)) ) \quad \forall i \in I. \end{aligned}$$

The weights $\mathbf {W}_t$ are computed based on the principles of an Ensemble Kalman Filter. As a result, each latest solution $\mathbf {Z}_t(:, i)$ accounts for all previously integrated data ($\mathbf {d}_0$ to $\mathbf {d}_{t-1}$). Integrating production data does not just result in updates of already extracted blocks (reconciliation). Neighbouring blocks, scheduled to be mined in the near future, are adjusted as well. The interested reader is referred to Wambeke and Benndorf (2017) for a detailed explanation of the algorithm.

**Fig. 1:**
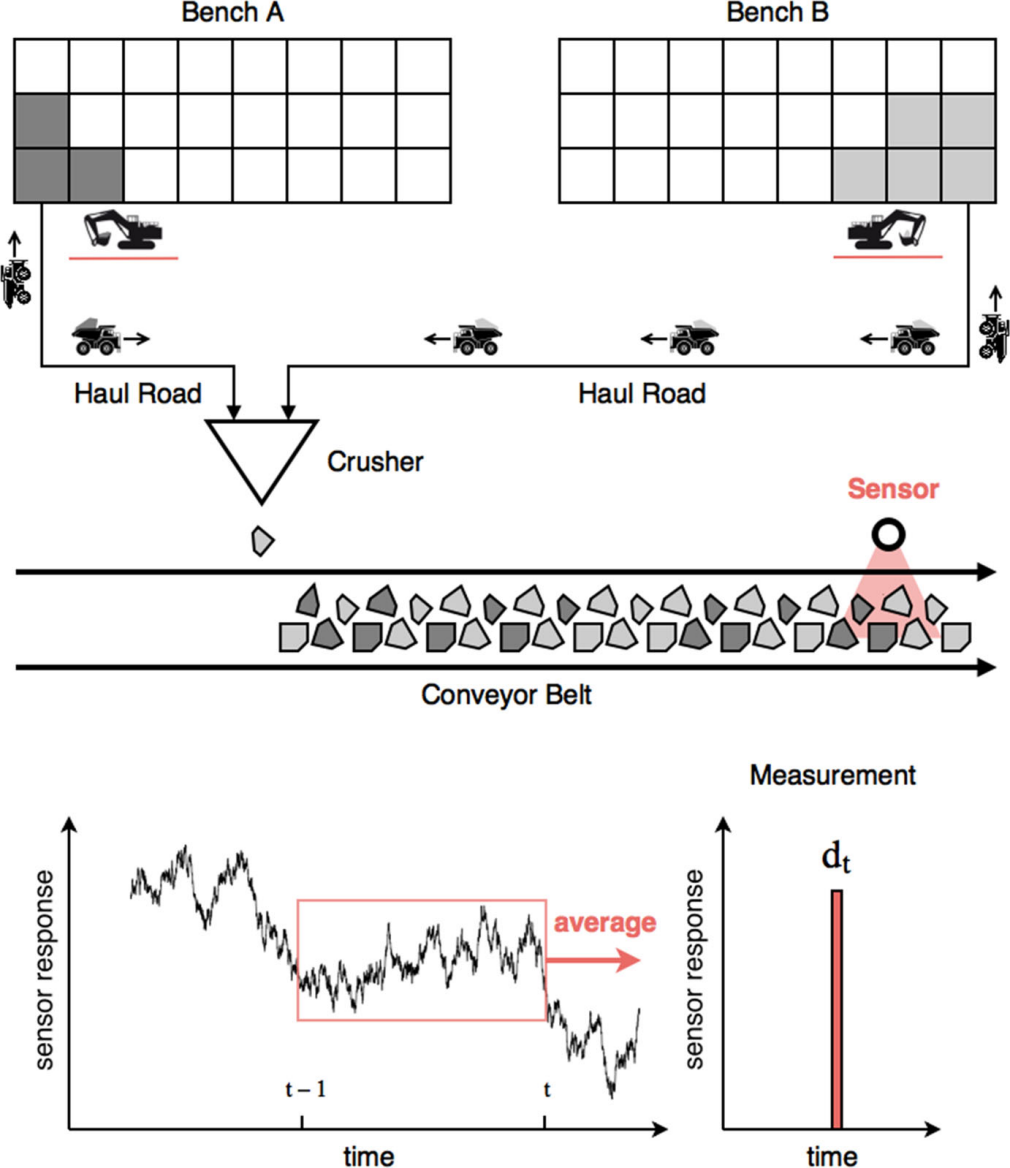
Monitoring setup in an open pit mining operation—the coloured blocks refer to material that has been extracted during the studied time interval ($t-1$ to *t*)

In order to apply the approach, a set of *I* estimates $\mathcal {A}_t(\mathbf {Z}_{t-1}(:, i))$ needs to be generated and the corresponding measurements $\mathbf {d}_t$ are to be collected. At the start of each time interval $[t-1, t]$, a set of estimates $\mathcal {A}_t(\mathbf {Z}_{t-1}(:, i))$ is obtained by individually propagating the GC model realisations through a forward simulator $\mathcal {A}_t$. The forward simulator is a virtual model of the actual operation and describes which blocks are extracted, processed and measured between $t-1$ and *t*. This forward prediction step is essential in linking specific observations $\mathbf {d}_t$ to their constituent blocks (Wambeke and Benndorf 2017). Figure 1 shows an example of a monitoring system in an open pit mining operation. In this example, material is extracted from two benches with different local production rates. After hauling, material is tipped into a comminution circuit. Inside the comminution circuit, a sensor continuously records the properties of the crushed material. As soon as a time period ends, an average measurement $\mathbf {d}_t$ is computed based on the recorded sensor response.

The performance of the algorithm directly depends on following three system parameters. (i) The measurement volume $V_t$ equals the amount of material that has been characterised between $t-1$ and *t*. The measurement volume is controlled by adjusting the interval duration ($[t-1,t]$) and the local production rates (Fig. 1). (ii) The blending ratio $R_{t,X}$, formulated as a percentage, defines how much material in the measurement volume originates from bench *X* (should sum up to one). The blending ratios are determined by the local production rates. (iii) The measurement error $E_t$ represents the accuracy of the applied sensor. The objective of this paper is to study the influence of these three system parameters on the overall performance of the algorithm. A total of 125 experiments are conducted to derive some general recommendations to optimally operate the monitoring system. This contribution is to be regarded as the sequel on a previous publication (Wambeke and Benndorf 2017).

## Methodology

### General Setup

The 125 experiments represent a mining operation with two extraction zones located in different benches. Material streams originating from both benches are blended, and inaccurate observations are made (Fig. 1). The experiments are conducted using the following boundary conditions; (i) the true, but unknown geological reality does not change across the experiments, (ii) the prior model of this geological reality is the same across experiments, (iii) the global production rate is assumed constant across all timesteps and experiments, (iv) the duration of each experiment is the same. Even though exactly the same material is excavated, processed and measured during the course of each experiment, the configuration of the monitoring system significantly influences the end results. From this point forward, the measurement volume $V_t$ is expressed in number of blocks. Measurement volumes of 8, 16, 32, 48 and 64 blocks are considered. Note, the production rate remains constant at 8 blocks per time unit.

The blending ratio $R_{t, A}$ defines how many of the blocks in the measurement volume originate from bench *A*. Consequently, the remaining blocks originate from bench *B*. For example, a blending ratio of $75\%$ and a measurement volume of 48 blocks prescribe that each single blended measurement is composed of 36 blocks from bench *A* ($N_A$) and 12 blocks from bench *B* ($N_B$). Blending ratios of 100, 87.5, 75, 62.5 and 50$\%$ are studied. The measurement error $E_t$, defined as the standard deviation of a zero mean normal distribution, represents the precision of the applied sensor. Measurement errors of 0.05, 0.25, 0.5, 0.75 and 1.00 are considered. The reported standard deviations define the measurement error on a scale of a single block (discussed later on).

From this point forward, units of the modeled attributes are omitted to stress their synthetic nature.

### Geology

#### True But Unknown Field

During the course of an experiment, a true but unknown geological state is sampled twice. First, small point samples are collected on both benches prior to the construction of the GC model (exploration phase). Second, observations are made on a blend of multiple blocks, which do not necessarily originate from the same bench (operational phase). To account for both scales of support correctly, two representations of the true, but unknown state are constructed. Both a high-resolution point and lower-resolution block reference field characterise the true but unknown geological state in two benches.

The high resolution point representation is constructed on two discretised grids, each with $300 \times 300$ cells of size 1 m $\times$ 1 m. The origins of bench *A* and *B* are located in (0 m, 0 m) and (1000 m, 1000 m). The two reference fields are generated using a random field simulation algorithm (sequential Gaussian simulation). Attribute values are drawn from a standard normal distribution (centred around 0). The correlation between attribute locations is described using an isotropic exponential function with a variance of one and a range of 100 m. Each attribute value thus reflects the direction ($+/-$) and magnitude of a deviation from a global mean.

**Fig. 2:**
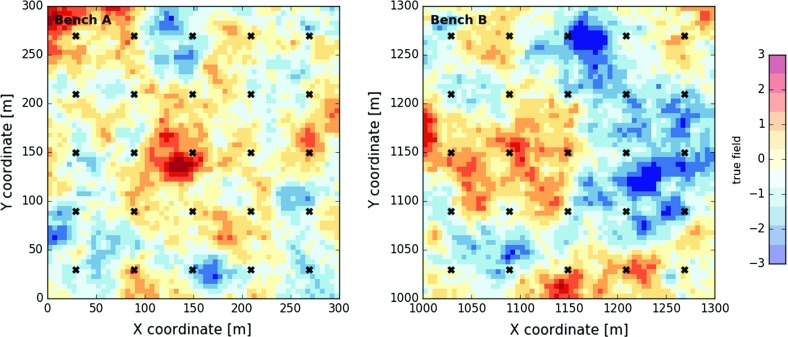
Block representation of the true, but unknown geological state in bench *A* (left) and bench *B* (right)—$60 \times 60$ blocks of size 5 m $\times$ 5 m color-coded according to their average block value—black crosses indicate the locations of the sampling points collected on a 60 m $\times$ 60 m exploration grid—point samples have a support of 1 m $\times$ 1 m

The lower-resolution block representation is constructed on two discretised grids, each with $60 \times 60$ cells of size 5 m $\times$ 5 m. This second set of reference fields is obtained from reblocking the previous point fields. Values of $5 \times 5$ 1 m $\times$ 1 m cells are averaged and assigned to larger 5 m $\times$ 5 m blocks. The resulting block reference field in bench *A* has an average of $-\,0.145$ ($-\,0.432$) and a variance of 0.659 (1.012). The 5 and $95\%$ quantiles are $-\,1.444$ ($-\,2.068$) and 1.181 (1.209). The values between brackets refer to the statistics computed for bench *B*. As expected, the variability decreases due to reblocking. The final block reference fields of bench A and B are displayed in Fig. 2.

#### Prior Model

Two sets of field realisations are used throughout to characterise the spatial variability and geological uncertainty of the study environment in benches *A* and *B*. Prior to the operation, exploration data is being collected on a 60 m $\times$ 60 m sampling pattern. This sampling density results in two prior sets of realisations with a realistic level of uncertainty and error.

**Fig. 3:**
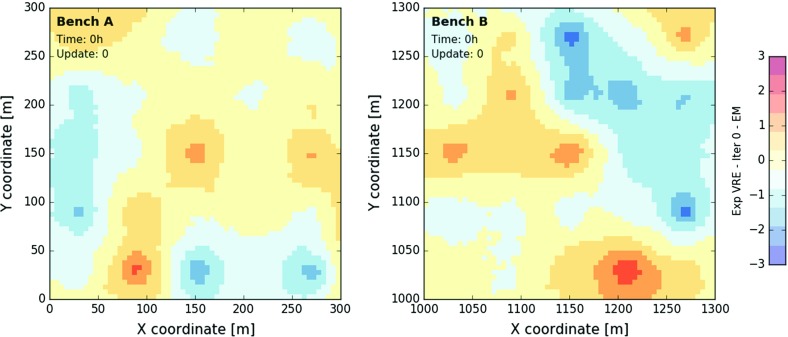
Bench *A* (left) and bench *B* (right) with 60 $\times$ 60 blocks of 5 m $\times$ 5 m color coded according to the computed mean field

Two prior sets of 100 realisations are generated on discretised grids with $300 \times 300$ cells of size 1 m $\times$ 1 m using a sequential Gaussian simulation algorithm (same as before). A total of 50 sampling points are provided as conditioning data (25 per grid). The covariance model of the true state is assumed to be known and was not inferred from the sampling points. Once obtained, the 100 point simulations are reblocked onto a grid with 60 $\times$ 60 blocks of size 5 m $\times$ 5 m. Only the block realisations are further considered (one reference field $\mathbf {Z}^*$ and 100 prior block realisations $\mathbf {Z}_{0}(:,i)$ per bench). All previous point simulations were just an aid to this end. Figure 3 displays the mean field (EM) computed from the 100 prior realisations. The mean fields of benches *A* and *B* are considerably smoother than the true state (compare Figs. 2, 3). The general larger-scale patterns are nevertheless reproduced.

### Experimental Scenarios

**Fig. 4:**
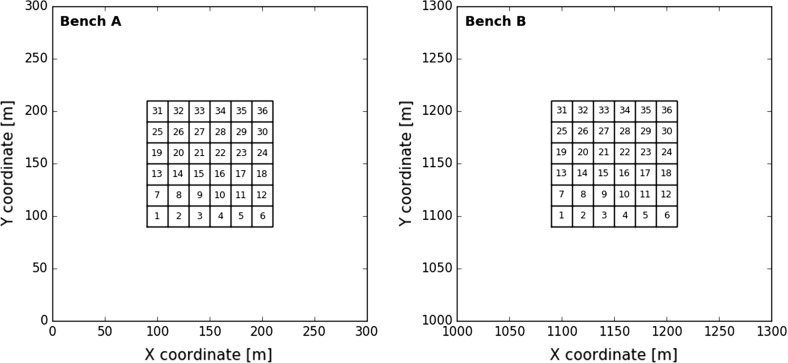
Extraction zones (120 m $\times$ 120 m) and digging blocks (20 m $\times$ 20 m) in bench *A* (left) and bench *B* (right)—the numbers indicate the order in which digging blocks are extracted from a bench

During each experiment, a total of 1152 blocks are blasted, excavated, processed and monitored. The corresponding blocks originate from two possible extraction zones in benches *A* or *B* (Fig. 4). Each experiment terminates after 144 h. This is the time needed to remove all blocks from the designated extraction zones assuming a constant global production rate of eight blocks per hour.

#### Composition Measurement Volume

The extraction zones are further subdivided into 36 20 m $\times$ 20 m digging blocks. The numbers in Fig. 4 indicate the order in which digging blocks are extracted from a bench. The same sequential pattern is used to extract 16 GC blocks (5 m $\times$ 5 m) from each digging block (from left to right in a row, move row from bottom to top). Both extraction patterns are combined to construct two nested queues (one for each bench). Each nested queue uniquely defines the order in which the smaller GC blocks are removed from a particular bench. The order of extracting blocks from each bench does not change across the 125 experiments.

**Fig. 5:**
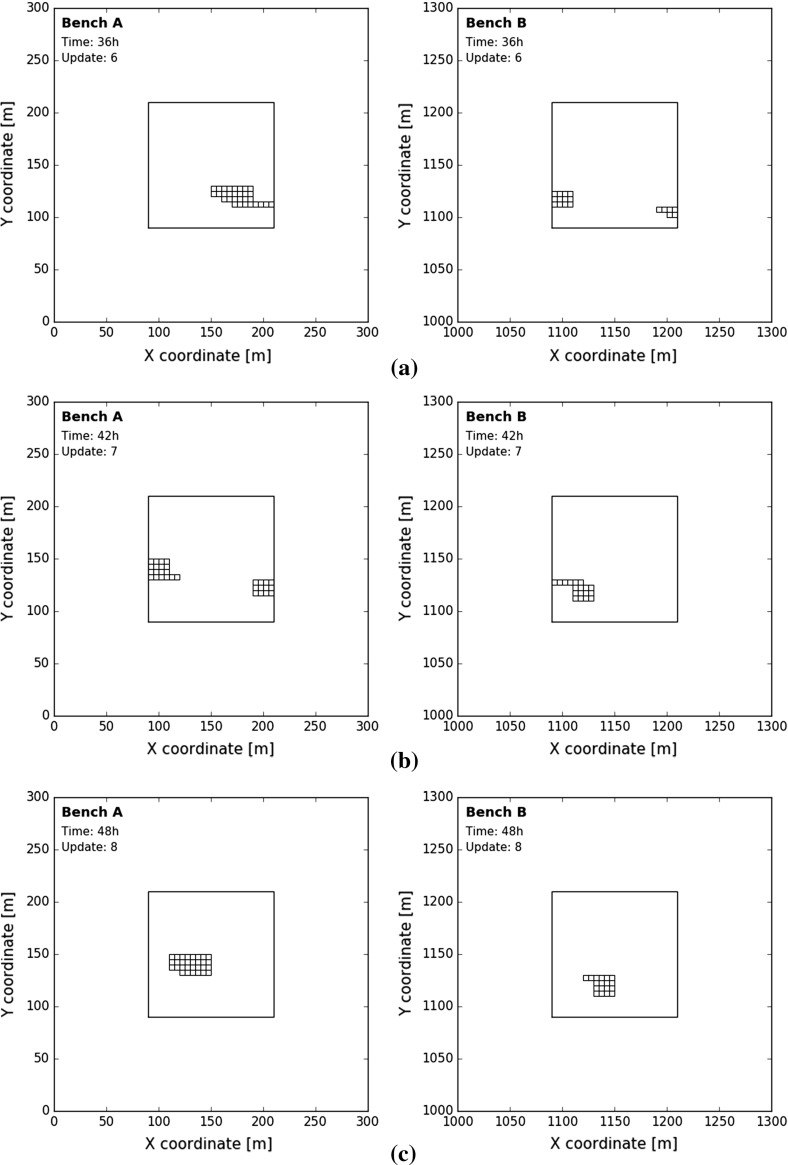
Blocks from bench *A* (left) and bench *B* (right) in blend at 36 h (**a**), 42 h (**b**), 48 h (**c**)—measurement volume of 48 blocks and blending ratio of $62.5\%$—blending scenario 44E

The specific time when a block is extracted, processed and measured does change across experiments. Algorithm 1 describes the composition of the blend at discrete timesteps given a measurement volume $V_t$ and a blending ratio $R_{t,A}$. A blend is a collection of blocks passing the sensor between $t-1$ and *t*. Note that the blending ratio $R_{t,A}$ is changed to $100-R_{t,A}$ when 576 blocks are extracted (lines 19–20, Algorithm 1). This is to ensure that both extraction zones are fully depleted at the end of each experiment. Figure 5 displays the composition of the blend at 36, 42 and 48 h given a measurement volume of 48 blocks and a blending ratio of $62.5\%$. Occasionally, scheduled blocks are dispersed across the bench (Fig. 5a). Table 1 provides an overview of the 25 different blending scenarios. Note that the measurement volume determines the time between updates and, therefore, also the total number of possible updates within each experiment.
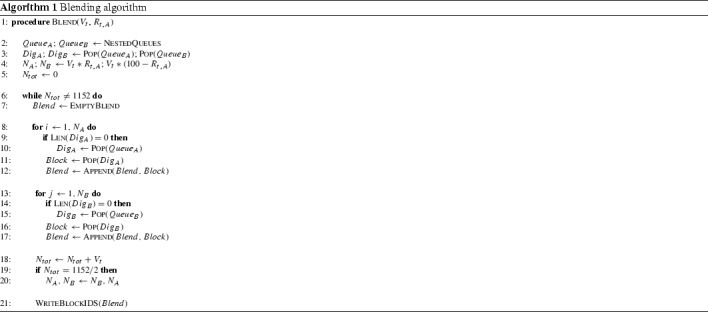

**Table 1:** Blending scenarios defined by measurement volumes $V_t$ and blending ratios $R_{t,A}$—Upd. is number of updates within an experiment, dt is time between updates—$N_A$ and $N_B$ are the number of blocks from bench *A* and bench *B* constituting a blended measurement—the *E* in the experiment code refers to an unassigned measurement error

Exp.	$V [\#]$	$\hbox {Upd.} [\#]$	dt[h]	$R [\%]$	$N_A [\#]$	$N_B [\#]$
11E	8	144	1	100	8	0
21E	16	72	2	100	16	0
31E	32	36	4	100	32	0
41E	48	24	6	100	48	0
51E	64	18	8	100	64	0
12E	8	144	1	87.5	7	1
22E	16	72	2	87.5	14	2
32E	32	36	4	87.5	28	4
42E	48	24	6	87.5	42	6
52E	64	18	8	87.5	56	8
13E	8	144	1	75	6	2
23E	16	72	2	75	12	4
33E	32	36	4	75	24	8
43E	48	24	6	75	36	12
53E	64	18	8	75	48	16
14E	8	144	1	62.5	5	3
24E	16	72	2	62.5	10	6
34E	32	36	4	62.5	20	12
44E	48	24	6	62.5	30	18
54E	64	18	8	62.5	40	24
15E	8	144	1	50	4	4
25E	16	72	2	50	8	8
35E	32	36	4	50	16	16
45E	48	24	6	50	24	24
55E	64	18	8	50	32	32

### Forward Simulator and Sensor Response

The forward simulator is constructed based on the assumption that an actual measurement $\mathbf {d}_t$ would result from a time-averaged sensor response characterising the composition of the blend. To mimic this behavior, the forward simulator translates a set of realisations $\mathbf {Z}_{t-1}(:, i)$ into a set of estimated observations $\mathcal {A}_t(\mathbf {Z}_{t-1}(:, i))$ by averaging a selection of block values. Blocks are selected with the aid of Algorithm 1. The values to be averaged are obtained from the relevant GC model realisation. The time between consecutive runs (time between $t-1$ and *t*) is determined by the measurement volume (Table 1).

Because of the synthetic nature of the experiment, a second forward simulator $\mathcal {A}^*_t$ is built to mimic the behaviour of a real monitoring network. This simulator generates inaccurate, but true observations $\mathbf {d}_t$ based on the reference field $\mathbf {Z}^*$ ($\mathbf {d}_t = \mathcal {A}^*_t(\mathbf {Z}^*)$). The previous constructed simulator $\mathcal {A}_t$ is adjusted to suit this purpose. Two main modifications are made. First, the reference field $\mathbf {Z}^*$ is propagated through the simulator and not one of the realisations $\mathbf {Z}_{t-1}(:,i)$. Second, random noise is added onto the individual block values. The random noise is drawn from a zero mean normal distribution with a standard deviation $E_t$, previously referred to as the measurement error (in order to compare results across experiments, the same random seed is used throughout). Once the noise is added, a time-averaged measurement $\mathbf {d}_t$ is computed. For further reference, the accuracy of the time-averaged measurement amounts to $E_t$/$\sqrt{V_t}$ ($V_t$, in number of blocks).

A total of 125 experiments are conducted based on different *VRE* combinations (5x5x5). Each experiment is given a unique three digit code. The first two digits refer to the blending scenario as indicated in Table 1. The third digit will indicate the precision of the applied sensor (1 for an *E* of 0.05, 5 for an *E* of 1.00).

## Assessment Statistics

The raw data of the 125 experiments are further processed. The root mean square error (RMSE), is computed on several subsets of data to address the following questions. (i) Does the GC model improve over time? (ii) Do predicted measurements improve within a fixed 144 h window? (iii) Do predicted measurements improve within future moving 24 h windows?

The computed RMSEs are subdivided into two groups. (i) Field errors, derived from GC models, are used to answer the first question. (ii) Production errors, computed based on predicted measurements, are used to address the second and third question. Experiment 223 is discussed in greater detail to elucidate the approach (measurement volume of 16 blocks, blending ratio $87.5\%$, measurement error of 0.50).

### Field Errors

**Fig. 6:**
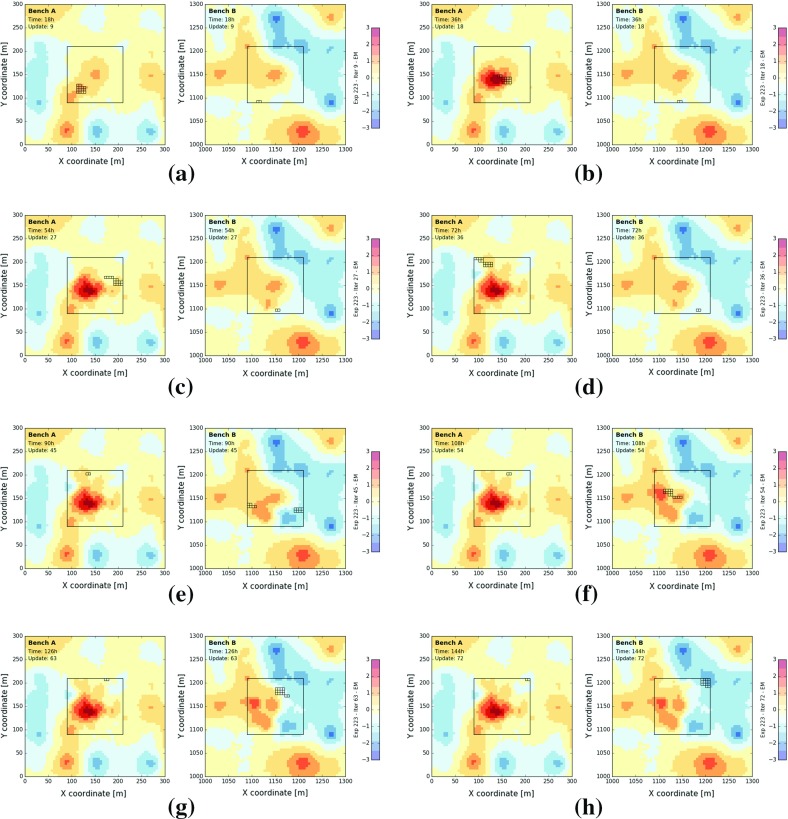
Mean field in bench *A* (left) and bench *B* (right) at 18 h (**a**), 36 h (**b**), 54 h (**c**), 72 h (**d**), 90 h (**e**), 108 h (**f**), 126 h (**g**), 144 h (**h**)—experiment 223, measurement volume of 16 GC blocks, blending ratio of $87.5\%$ and measurement error of 0.5

Figure 6 displays how the mean fields of bench *A* and *B* change through time when assimilating time-averaged sensor responses. The 16 smaller rectangles demarcate the blocks, which were extracted, blended and measured during the considered time interval. A comparison of Fig. 6a–h with the prior mean field (Fig. 3) and the true state (Fig. 2) indicates that the mean field continuously improves in and around both extraction zones.

**Fig. 7:**
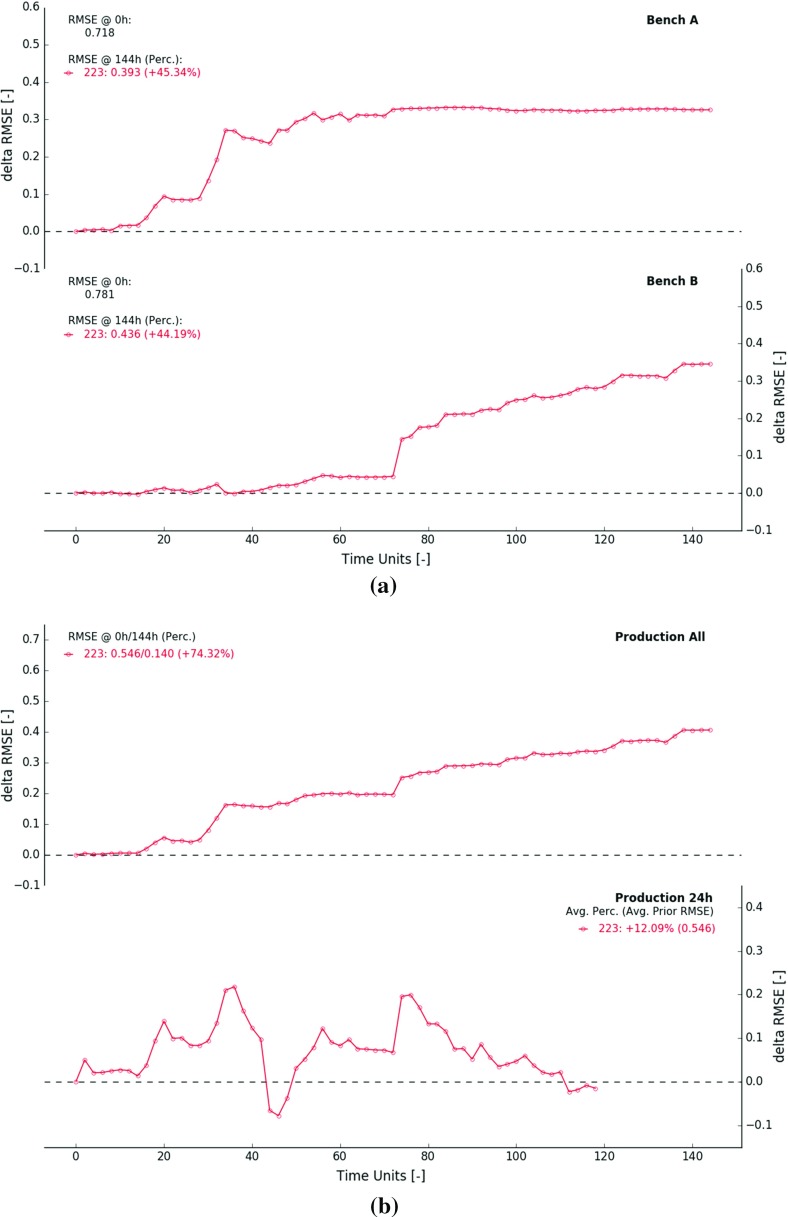
$\Delta$RMSE as a function of time—results from experiment 223 are displayed. **a** $\Delta$RMSE computed over the extraction zones in bench *A* (top) and bench *B* (bottom), **b** $\Delta$RMSE computed for predicted measurements within fixed 144 h time window (top) and a moving 24 h future outlook (bottom)

A more quantitative measure, such as the RMSE is computed to describe the overall deviation at time *t* between true ($\mathbf {Z}^*(n)$) and estimated block values ($E\big [ \mathbf {Z}_t(n, :) \big ]$)

2 $$\begin{aligned} \hbox {RMSE}_t = \sqrt{\frac{1}{N}\sum _{n=1}^{N} \bigg ( \mathbf {Z}^*(n) - E \big [\mathbf {Z}_{t}(n,:) \big ]} \bigg )^2. \end{aligned}$$

Two time series of RMSE are computed, one for each extraction zone. In this case, *n* only iterates over the 576 blocks located inside one of both studied extraction zones (Fig. 4). The change in RMSE quantifies obtained improvements (positive) or deteriorations (negative) relative to time 0

3 $$\begin{aligned} \Delta \hbox {RMSE}_t = \hbox {RMSE}_0 - \hbox {RMSE}_t. \end{aligned}$$

Ideally, the $\Delta$RMSE increases as more measurements are incorporated. Figure 7a displays the evolution of the $\Delta$RMSE through time. The RMSE of the prior model inside the extraction zones of bench *A* and *B* amounts to 0.718 and 0.781 respectively. Over the course of the experiment, the RMSE inside both extraction zones drops by about $45\%$ (45.34 and $44.19\%$).

Figure 7a indicates that the RMSE decreases approximately at a semi-constant rate during the first (0–72 h) and second half (72–144 h) of the experiment. The period of steep incline (faster reduction in RMSE) coincides with a larger local production rate (14 GC blocks per 2 h). Remind that the global production rate remains constant across the experiment (eight GC blocks per hour) and that the blending ratio is reversed after 72 h (lines 19–20, Algorithm 1). The somewhat volatile behaviour results from a larger impact of a few local improvements (Fig. 7a). For example, the exceptionally large reduction between 28 and 34 h at bench *A* originates from a correction of the anomalous central area (compare Fig. 6a, b). A similar exceptionally large reduction in RMSE occurs in bench *B* between 72 and 74 h (Fig. 7a, decreasing mean field near $x=1175$ m and $y=1125$ m, compare Fig. 6d, e).

### Production Statistics

**Fig. 8:**
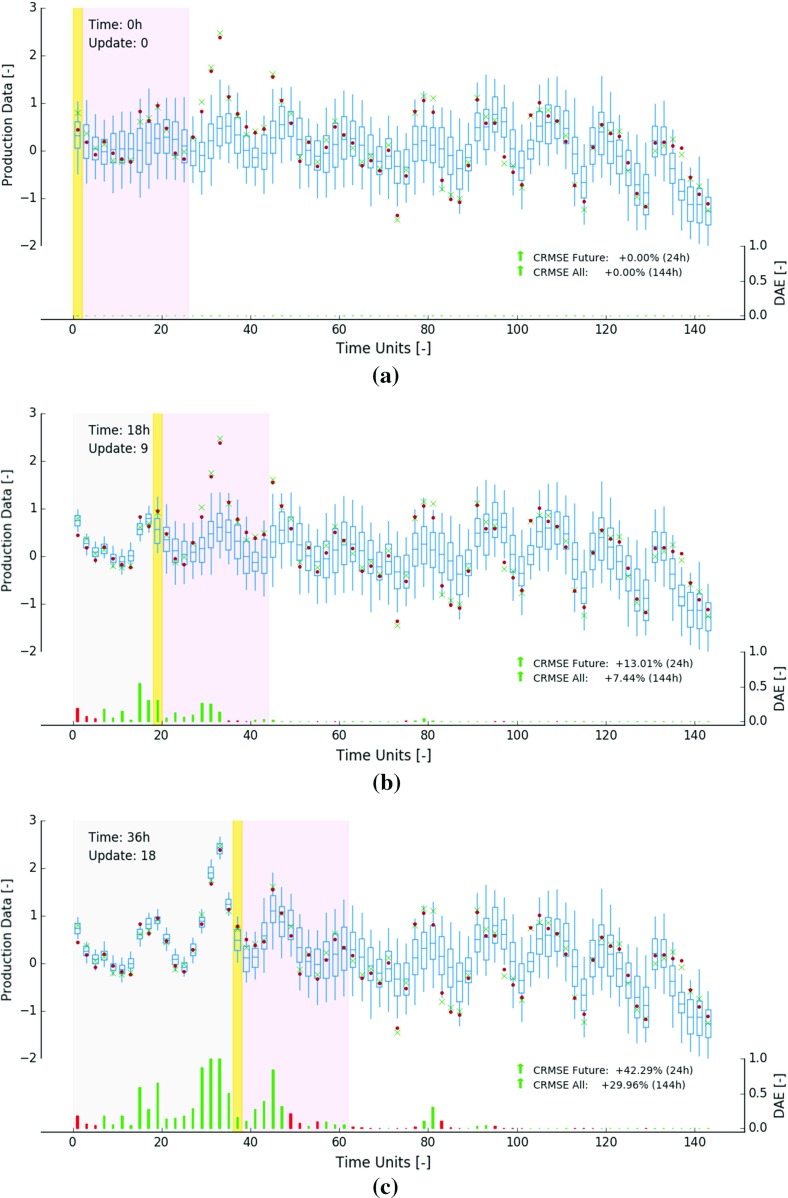
Predicted measurements (blue), time-averaged inaccurate sensor responses (green cross) and true, but unknown averages (red dot) at 0 h (**a**), 18 h (**b**), 36 h (**c**)

Figure 8a–c display predicted measurements $\mathcal {A}_t(\mathbf {Z}_{t-1}(:, i))$ (blue boxplots) computed from the GC model state at 0, 18 and 36 h. The figures further show time-averaged sensor responses ($\mathbf {d}_t$, green cross) and true, but unknown averages (red dots). Each plot displays the average composition of 72 2-h blends (16 blocks per blend). Some blends are already processed and measured (grey area), one is currently being characterised (yellow bar) and others still have to be extracted (right of yellow bar).

In practice, inaccurate measurements are not yet available ahead of the current time interval (no green crosses right of the yellow bar). Moreover, due to sensor inaccuracies, the true averages of the blends (red dots) are never known. In this synthetic experiment, the true averages of the blends are known and production assessment statistics can be computed. The bottom axes in Fig. 8 displays the differences in absolute error (DAE) between best estimates (blue horizontal lines in boxplots) and true averages (red dots) relative to time 0. For example, a DAE value of 0.5 indicates that the best estimate moved 0.5 units towards the true average. The bars are coloured green in case of improvements and red otherwise. Deteriorations (red bars) are relatively rare. Figure 8 further illustrates that GC model updates not only result in reconciled historic production data (grey area), but also improve future predictions (right of yellow bar).

Two sets of $\Delta$RMSE curves are computed to summarise improvements of predicted measurements. The first set is based on all available data within a fixed 144 h time window. The second set describes adjustments in error during the next 24 h (moving window, purple area right of yellow bar). The calculation proceeds as follows: (i) at time *t*, construct time interval—either $[0~\mathrm{h}, 144~\mathrm {h}]$ or $[t, t+24~\mathrm {h}]$, (ii) select predicted and real measurements within this time interval (iii) compute RMSE based on selected data (similar to Eq. 2), (iv) repeat ii–iii, but use prior predicted measurements (derived from GC model at time 0*h*), (v) compute $\Delta \hbox {RMSE}_t$, (vi) go to (i) and increase *t* to $t+dt$.

The two resulting curves are displayed in Fig. 7b. The RMSE of all prior predicted measurements (144 h window) amounts to 0.546 (Fig. 7b, top). Over the course of the experiment, the RMSE drops by about $74\%$. The two exceptionally large reductions between 28 and 34 h and 72–74 h originate from significant GC model corrections in bench *A* and *B* respectively (previously discussed). The cumulative improvement in error mainly results from the increasing number of reconciled historic measurements in the static 144 h window (Fig. 8, larger grey area).

Advancing a 24 h time interval while updating yields a different kind of behaviour (Fig. 7b, bottom). The $\Delta$RMSE fluctuates around a constant value. At every point in time, the sample set is composed of an equal number of future predicted measurements, all derived from unsampled scheduled GC blocks. The RMSE in future 24 h windows drops on average by about $12\%$. No results are displayed from 120 h onward due to incomplete 24 h windows.

## Results

This section elaborates on the effects of variations in either the measurement volume, blending ratio or measurement error.

### Measurement Volume

**Fig. 9:**
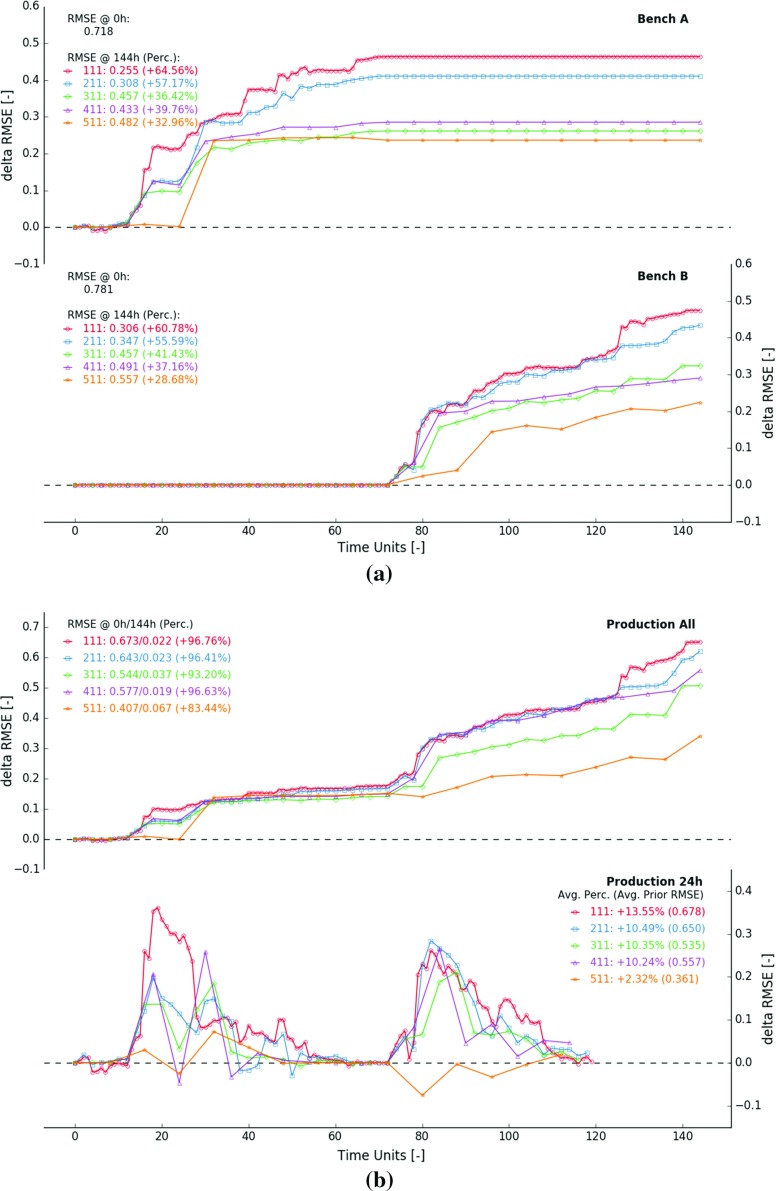
$\Delta$RMSE in function of time—measurement volumes amount to 8 (111), 16 (211), 32 (311), 48 (411) and 64 (511) blocks—blending ratio and measurement error are fixed ($100\%$ and 0.05). **a** $\Delta$RMSE computed over the extraction zones in bench *A* (top) and bench *B* (bottom), **b** $\Delta$RMSE computed for predicted measurements within fixed 144 h time window (top) and a moving 24 h future outlook (bottom)

Figure 9 displays results from five selected experiments, all conducted using a blending ratio of $100\%$ and a measurement error of 0.05. The measurement volume increases from 8 to 16, 32, 48 and 64 blocks. Figure 9a displays how the RMSE changes in function of time inside the extraction zones of benches *A* and *B*. An eight times larger measurement volume results in approximately half of the initial RMSE reduction. The 144 updates in experiment 111 reduce the RMSE in bench *A* and *B* with 0.464 ($64.56\%$) and 0.475 ($60.78\%$). In comparison, the 18 updates in experiment 511 result in a RMSE reduction of 0.237 ($32.96\%$) and 0.224 ($28.26\%$). The parabolic reduction in RMSE reflects the exceptionally large significance of a few local improvements. These significant improvements occur when the algorithm detects and corrects local anomalies. When excavating a new area, local anomalies are generally detected and corrected early on. The other updates merely result in smaller incremental improvements.

Larger measurement volumes apparently lead to fewer, but more significant updating events. In experiment 111, five large updating events occur improving the model of bench *A* (Fig. 9a top, $t={16, 18, 39, 40, 47}$ h). The five updates yield individual improvements ranging from 0.030 to 0.098. Combined, they account for more than half of the total obtained improvement (0.265 of the total 0.464). Contrarily, the fourth update (32 h) in experiment 511 is solely responsible for a reduction in RMSE of 0.233 (Fig. 9a, top). This single update is almost entirely responsible for the final obtained improvement in bench *A*. Similar observations can be made in bench *B*. The top graph of Fig. 9b displays the $\Delta$RMSE curves derived from predicted measurements and true averages (Ref. Fig. 8). Historic and future predicted measurements are jointly considered within a fixed 144 h time window (entire experiment duration). The overall reduction in RMSE decreases with an increase in measurement volume ($\Delta$RMSE, from 0.651 in exp. 111 to 0.340 in exp. 511). However, some caution is needed in interpreting production related results. As measurement volumes grow larger, the RMSE of predicted measurements is lower to start with ($\hbox {RMSE}_0$, 0.673 in exp. 111, 0.407 in exp. 511). As a result, the relative improvements barely vary across the experiments. During most experiments, the RMSE drops by about $95\%$.

**Fig. 10:**
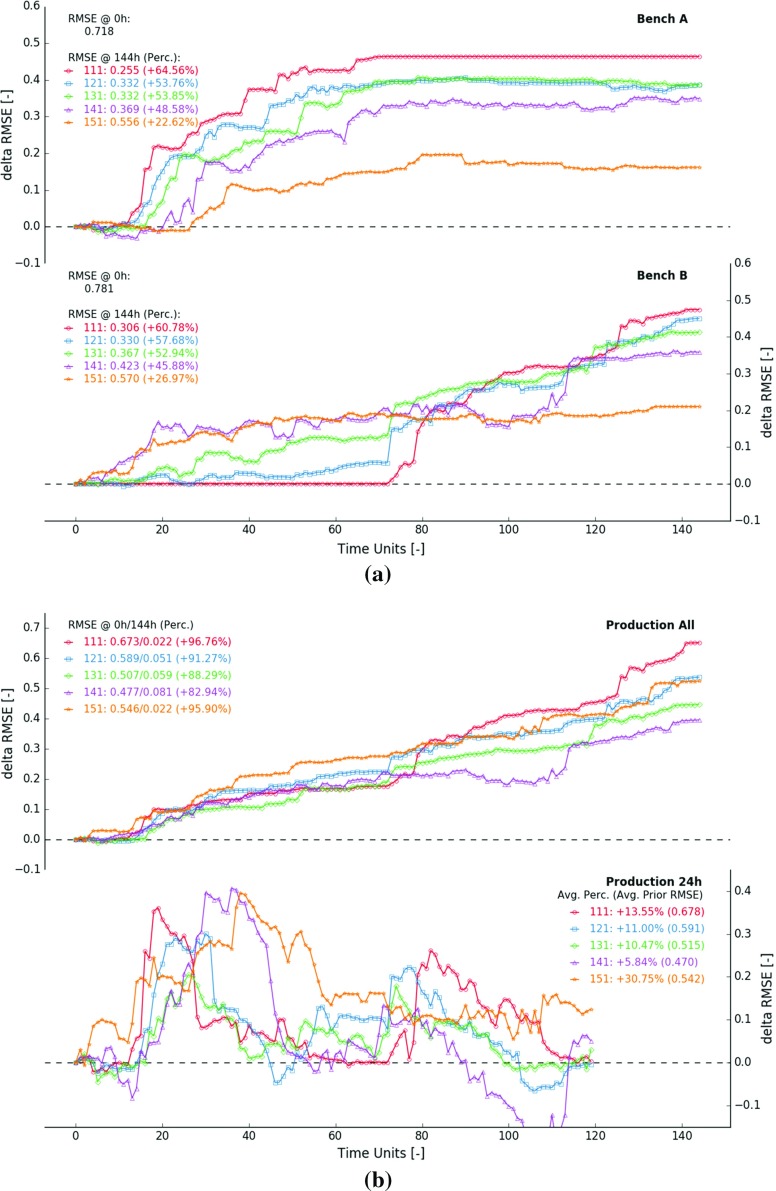
$\Delta$RMSE in function of time—blending ratios amount to $100\%$ (111), $87.5\%$ (121), $75\%$ (131), $62.5\%$ (141) and $50\%$ (151)—measurement volume and measurement error are fixed (8 blocks and 0.05). **a** $\Delta RMSE$ computed over the extraction zones in bench *A* (top) and bench *B* (bottom). **b** $\Delta$RMSE computed for predicted measurements within fixed 144 h time window (top) and a moving 24 h future outlook (bottom)

The bottom graph of Fig. 10 depicts improvements in future predicted measurements (next 24 h). The $\Delta$RMSE curves describe at time *t*, the reduction in error of upcoming predicted measurements in the time interval $[t, t+24~\mathrm{h}]$. Figure 9b illustrates that improvements diminish with larger measurement volumes. In experiment 111, the RMSE decreases on average by $13.55\%$. These relative improvements drop to $2.32\%$ when the measurement volume increases from 8 to 64 blocks (exp. 511). Note that the average prior RMSE also reduces with a larger measurement volume (avg. $\hbox {RMSE}_0$, 0.678 in exp. 111, 0.361 in exp. 511).

### Blending Ratio

Figure 10 displays results from five selected experiments, all conducted using a measurement volume of eight blocks and a measurement error of 0.05. The blending ratio is gradually reduced from 100 to $50\%$ with steps of $12.5\%$. A lower blending ratio results in a lower reduction of the RMSE inside both extraction zones (Fig. 10a). During experiment 111, the eight blocks in the blend always originate from the same bench. This particular configuration yields improvements of 0.464 ($64.56\%$, bench *A*) and 0.475 ($60.78\%$, bench *B*). Improvements drop to about 0.162 ($22.62\%$, bench *A*) and 0.211 ($26.97\%$, bench *B*) when half of the blocks originate from the other bench (experiment 151).

When both areas contribute an equal number of blocks to the blend, it becomes harder to pinpoint the source of the detected deviation. The algorithm updates more cautiously leading to a longer sequence of smaller incremental improvements. Local anomalies are easier to detect if the blended material originates from a single area. The observed difference between predicted and recorded measurements can be easily attributed to a single area with a higher level of confidence. Hence, the algorithm corrects more aggressively.

The corresponding larger blending ratio further result in abrupt changes in local production rates (lines 19–20, Algorithm 1). These abrupt changes influence the overall shape of the $\Delta$RMSE curves. Periods of steep incline coincide with larger local production rates (e.g. exp. 121—bench *A*, seven blocks per hour between 0 and 72 h). Lower local production rates result in more gentle slopes (e.g. exp. 121—bench *A*, one block per hour between 72 and 144 h).

Figure 10b displays the $\Delta$RMSE curves derived from all predicted measurements within a static 144 h time window (top graph). As expected, the prior RMSE generally decreases with a lower blending ratio ($\hbox {RMSE}_0$, 0.673 in exp. 111, 0.477 in exp. 141). Lower ratios often result in a lesser degree of correlation between the blended blocks. More extreme block values (difficult to estimate) are likely to be averaged out, as are their larger associated errors. If the blocks in the blend originate from a single area (large blending ratio), their corresponding values are going to be correlated. Extreme values are no longer averaged out, nor are their associated larger errors.

The overall reduction in RMSE tends to be larger in experiments with larger blending ratios ($\Delta$RMSE of 0.651 in exp. 111, 0.396 in exp. 141). Generally, the performance improves with a lesser amount of blending. The differences expressed in terms of relative improvements are less significant. The RMSE drops between 82 and $97\%$. Changes in blending ratio do not always fully explain the observed differences. In order to understand improvements in future predicted measurements (next 24 h), the more detailed underlying mining schedules need to be analysed instead. Two specific features are of importance. (i) A lower degree of correlation between blended blocks leads to a less informative time-averaged sensor response. The algorithm updates more cautiously. The errors in the GC model remain relatively high. Errors in subsequently derived future predictions do not significantly change (negative effect associated with lower blending ratios). (ii) A lower distance between extracted and scheduled blocks result in better informed future predictions (positive effect associated with lower blending ratios).

Two mining schedules are compared to demonstrate the interplay between both features. All eight blocks, extracted at 24 h according to schedule 11E, originate from a single 20 m $\times$ 10 m rectangular area (Fig. 4, top half digging block 12). The eight blended blocks will be correlated. The 192 blocks, scheduled to be extracted within the next 24 h, are located in digging blocks 13–24 of bench *A* (Fig. 4). Only half of the blocks (digging blocks 13–18) lie within a distance of 20 m of the nearest mining face (horizontal line at $y=130$ m). In contrast, schedule 15E describes a simultaneous extraction from two mining faces; one at $y=130$ m in bench *A*, the other at $y=1130$ m in bench *B* (Fig. 4). The next 192 scheduled blocks all fit within the next row of digging blocks in either bench *A* or *B*. Consequently, all 192 blocks lie within a distance of 20 m from the nearest mining face. However, schedule 15E results in a lower degree of correlation between the blended blocks. The four blocks extracted from bench *A* will hardly be correlated with the four blocks from bench *B*.

Figure 10b illustrates how both opposing features impact performance. During experiment 111, the RMSE drops on average by $13.55\%$ (Fig. 10b, bottom). The average reduction in RMSE in experiment 151 more than doubles ($30.75\%$). Despite the additional extraction point, the performance improves due to a reduced average distance between extracted and scheduled blocks (feature 2 is dominant). These types of performance gains are untenable when average distances increase. The degree of correlation between the blended blocks becomes the impetus behind the observed RMSE reduction (feature 1). During experiment 121, 131 and 141, the RMSE drops with 11.00, 10.47 and $5.84\%$.

Experiment 111 further demonstrates that large errors in future predicted measurements are not necessarily corrected on time (Fig. 10b, bottom, between 60 and 72 h). During the last 12 h of excavation in bench *A*, the majority of predicted measurements in the next 24 h are based on block estimates from bench *B*. Until the processing of material from bench *B* actually commences, there is no way to know that the GC model needs to be locally corrected. Hence, very few improvements are observed between 60 and 72 h ($\Delta \hbox {RMSE} \approx 0$). As soon as data become available, the GC model is updated and the corresponding future predictions are corrected. No such unpleasant surprises occur during experiment 151. Future predicted measurements are always corrected on time. Since both benches are extracted simultaneously, scheduled blocks are continuously better informed due to the proximity of localised production data (measurements attributed to one or multiple source areas). Hence, the RMSE drops consistently around $30\%$ within all 24 h production windows (Fig. 10b).

### Measurement Error

**Fig. 11:**
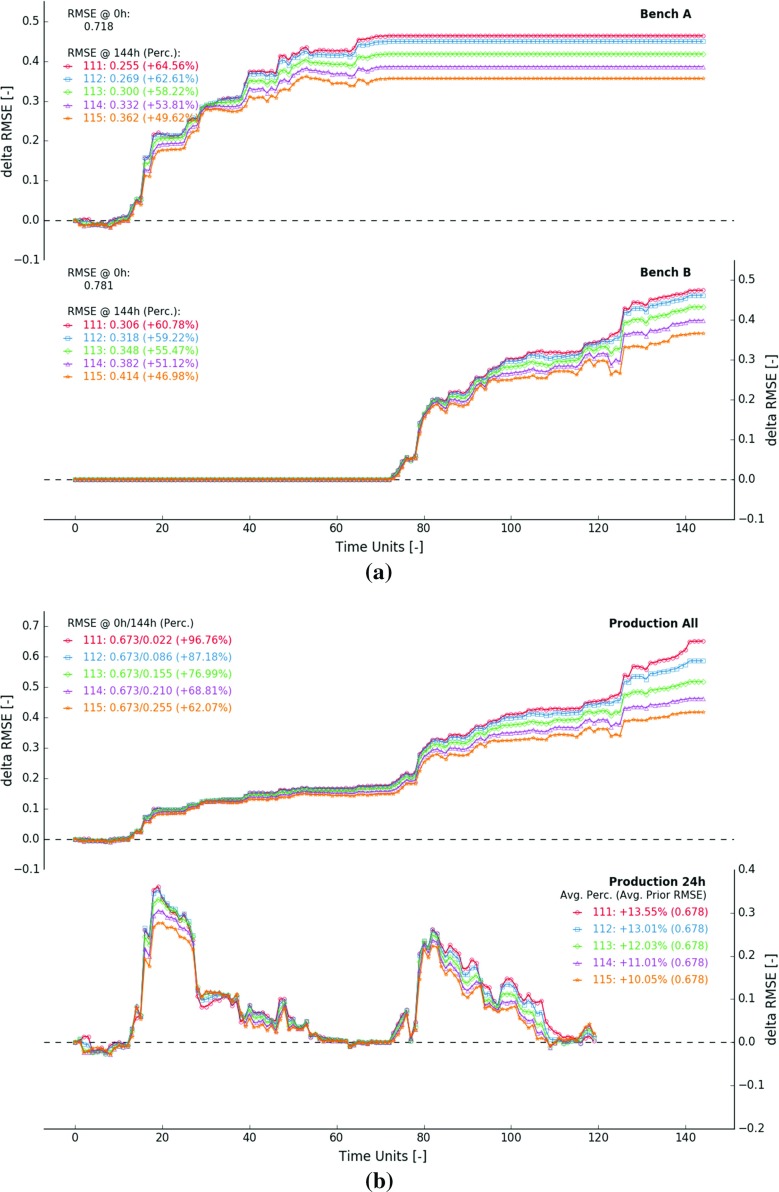
$\Delta$RMSE in function of time—measurement errors amount to 0.05 (111), 0.25 (112), 0.50 (113), 0.75 (114), 1.00 (115)—measurement volume and blending ratio are fixed (8 GC blocks and $100\%$). **a** $\Delta$RMSE computed over the extraction zones in bench *A* (top) and bench *B* (bottom). **b** $\Delta$RMSE computed for predicted measurements within fixed 144 h time window (top) and a moving 24 h future outlook (bottom)

Figure 11 displays results from five selected experiments, all conducted using a measurement volume of 8 GC blocks and a blending ratio of $100\%$. Sensors with measurement errors of 0.05, 0.25, 0.50, 0.75 and 1.00 are used. The selected standard deviations define the measurement error $E_t$ on a scale of a single GC block. To put the given numbers in perspective, the standard deviations of all prior GC block estimates vary between 0.284 (near exploration holes) and 1.033. The accuracy of the time-averaged sensor response characterising 8 GC blocks then amounts to $E_t$/$\sqrt{8}$.

Figure 11a displays how the RMSE changes in function of time inside the extraction zones of benches *A* and *B*. The algorithm updates more cautiously when the measurement error is larger. Individual smaller updates accumulate into lower final $\Delta$RMSE values (Fig. 11a). For example, the RMSE reduction observed over the course of experiment 111 amounts to 0.464 ($64.56\%$, bench *A*) and 0.475 ($60.78\%$, bench *B*). During experiment 115, the RMSE in bench *A* and *B* drops by 0.356 ($49.62\%$) and 0.367 ($46.98\%$), respectively. Despite the 20 times larger measurement error, the performance loss remains manageable.

Figure 11b displays the $\Delta$RMSE curves derived from all predicted measurements within a static 144 h time window (top graph). The blending ratio and measurement volume do not change across the five experiments, hence the constant prior RMSE (0.673). The performance drops considerably when increasing the measurement error. Conducting an experiment with a measurement error of 0.05 results in a $96.76\%$ RMSE reduction (exp. 111). The observed improvement drops to $62.07\%$ when employing a sensor with a measurement error of 1.00 (exp. 115).

The bottom graph of Fig. 11b illustrates that relative improvements in future predicted measurements (next 24 h) are not significantly influenced by the magnitude of the measurement error. In experiment 111, the RMSE decreases on average by $13.55\%$. Despite a twenty times larger measurement error, the average observed improvement during experiment 115 still amounts to $10.05\%$.

## Discussion

The aim of this section is to translate previous observations into three sets of operational recommendations.

### Objective 1: Maximise Error Reduction in GC Model

**Fig. 12:**
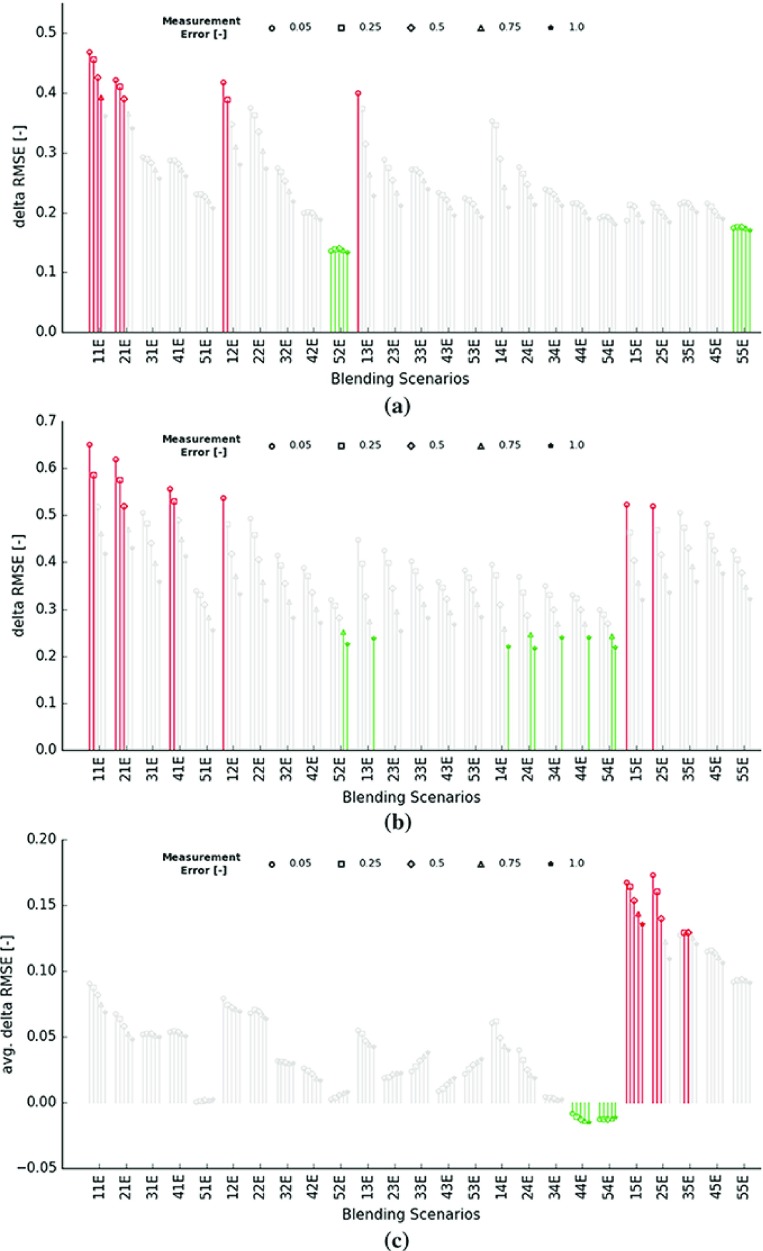
Reductions in RMSE—blending scenarios are indicated on the *x* axis (Table 1)—markers refer to the measurement error—10 best (red) and 10 worst (green) performing experiments are colored. **a** Error reduction in extraction zone *A* and *B* recorded at end of each experiment $(\Delta \hbox {RMSE}_A + \Delta \hbox {RMSE}_B)/2$. **b** Error reduction of predicted measurements in static 144 h window recorded at the end of each experiment. **c** Time-averaged error reduction of predicted measurements across all available 24 h windows, computed for each experiment

As soon as local production rates are unequal, one of the most effective measures to increase performance (in terms of maximising the RMSE reduction in the GC model) is to reduce the interval duration. If possible, local production rates should be adjusted such that the majority of the blended material originates from a single area. Investing in accurate sensors only seems to pay off when the interval duration is kept small. Figure 12a illustrates that variations in interval duration (time between $t-1$ and *t*) and/or sensor precision do have little to no impact if both areas contribute an equal number of blocks to the blend (Fig. 12a, 25 rightmost experiments).

### Objective 2: Optimise Reconciliation of Production Data

The second objective aims to minimise deviations between actual and predicted measurements ($\mathbf {d}_t - \mathcal {A}_t(\mathbf {Z}_{t-1}(:,i))$). Figure 12b displays, per experiment, the last recorded $\Delta$RMSE values computed from predicted measurements within static 144 h windows. Lowering the interval duration only seems to have a noticeable positive effect (larger RMSE reduction) if accurate sensors are applied and/or local production rates differ significantly (*E* of 0.05 or 0.25—*R* of 100 or $82.5\%$). Experiments with highly inaccurate sensors and converging local production rates (*R* of 75, 62.5 or $50\%$—*E* of 0.75 or 1.00) do seem to perform better as the measurement volume enlarges from 8 to 32 blocks. This is somewhat expected since the overall accuracy of recorded measurements ($E_t$/$\sqrt{V_t}$) increases as noisy sensor responses are averaged over larger volumes or periods of time (larger $V_t$). Opting for accurate sensors is another effective strategy to further reduce the RMSE (larger $\Delta$RMSE when *E* is lower). Adjusting the local production rates also affects the RMSE reductions. Results are better when production rates are either very alike or very different (blending ratio of 100, 82.5 and $50\%$, relates to underlying mining schedule—ref. previous discussion). Interestingly, the system parameters considered most optimal in terms of reconciled production data do not necessarily lead to the best GC model improvements (compare Fig. 12a, b).

### Objective 3: Maximise Error Reduction in Future Predictions

The average reduction in RMSE (avg. $\Delta$RMSE) needs to be maximised for optimal improvement of future predictions. Equal local production rates seem to lead to exceptional performances (Fig. 12c, blending ratio of $50\%$). As local production rates converge, distances between scheduled and extracted GC blocks decrease. Presumably, converging local production rates only result in a performance gain if the average distance is sufficiently low (feature 2). As long as this threshold is not reached, the performance drops due to the ever lower correlation between the blended blocks (feature 1). Essentially, in order to optimise future predictions, an optimal schedule has to be found (Sect. 6).

Figure 12c further illustrates that lower interval durations generally result in larger RMSE reductions. When interval durations are small, more accurate sensors definitely add value. The outcome of installing better sensors is less predictable as soon as interval durations start to increase.

### Practical Implications

Nearly all results have shown that shorter interval durations and lower measurement volumes improve algorithm performance, no matter the considered metric. Because of the complexity of the material handling process, it might be challenging to construct a forward simulator accurate enough to track material on a scale as small as eight blocks. It further would be wise to install by default the most accurate sensor available. First of all, a more accurate sensor significantly improves the reconciliation of production data (objective 2). Secondly, even in combination with medium measurement volumes (16 and 36 GC blocks), do GC models improve more significantly when accurate sensors are applied. In practice, it will be nearly impossible to control local production rates for the sake of improving the algorithm performance. Instead, local production rates are to be considered as external input and will very likely change over time. The previous results nevertheless can be used to predict how the algorithm performance will change as local production rates are adjusted.

## Conclusions

Recently, a new algorithm has been developed to update repeatedly the grade control model based on online data from a production monitoring system. The added value of the presented algorithm results from its ability to handle inaccurate observations made on blended material from two or more extraction points. A total of 125 artificial experiments are conducted to evaluate the influence of system parameters on the overall algorithm performance. Each experiment mimics a virtual mining operation with two extraction points. Over the course of a 144 h long experiment, 1152 blocks are excavated, processed and measured. Local production rates and interval durations vary across the experiments. Various sensors with a different precision are applied.

Results regarding GC model improvements are promising. Under fairly optimal conditions, the RMSE in the GC model drops around $60\%$. Optimal conditions occur when small operational volumes are extracted from a single location and characterised by a relatively accurate sensor (8 GC blocks, blending ratio of $100\%$, sensor precision of 0.05). Even when conditions are far from optimal, the RMSE in the GC models can still be reduced by about $20\%$.

The experiments further illustrate the outstanding reconciliation capabilities of the updating algorithm. The RMSE between historic predicted and actual measurements decreases between 46 and $97\%$ over the course of an experiment. Reconciliation improves when a more accurate sensor is applied. Larger RMSE reductions are observed when local production rates are sitting at either end of the spectrum (blending ratio of 100 or $50\%$). Interval duration does not significantly impact results. Improvements regarding future predicted measurements (next 24 h) turn out to be less robust against variations in system parameters. Average RMSE reductions vary between $-\,5$ and $+\,30\%$.

This contribution assumes that all assimilated data points are representative for their corresponding material streams. This is a rather strong assumption and needs to be verified for every practical application. Data points might not be representative for the following two reasons: (i) the sensor setup only measures a relatively small portion of a cross-sectional area (i.e. area of the material pile perpendicular to the direction of conveying), (ii) the time between measurements is too large (i.e. distance between measured cross-sections, parallel to the direction of conveying). The question of how many sensor readings are required to characterise the material stream representatively still has to be addressed.

More work should be conducted to determine the influence of the correlation between extracted and scheduled blocks. A single-value statistic must be devised describing the updating potential of a mining schedule at a discrete timestep. Three types of correlations must be considered; cross-correlations between recently excavated blocks, cross-correlations between near-term scheduled blocks and correlations between recently extracted and near-term scheduled blocks. All this information could possibly be captured in some sort of signal-to-noise ratio. A large ratio would predict a good amount of improvement in future predictions.

Future research should further focus on how the prior realisations impact local anomaly corrections later on. The empirical covariances lifted from the prior realisations largely determine the extent of updates in neighbouring block values. When the covariances between measurements and neighbouring blocks are overestimated, attribute values are corrected too aggressively, causing erroneous updates. A corrective localisation technique is integrated into the algorithm to prevent such behaviour (Wambeke and Benndorf 2017). Underestimated covariances on the other hand yield updates which are too modest. Certain local anomalies remain partly uncorrected. In reality, the correlation structure of the true field is obviously unknown. Several parallel updating tracks could be run simultaneously, all initiated with a specific set of prior realisations (each set could be generated using a different variogram model and/or simulation technique). Error statistics, collected during an initial training period, could eventually be compared to select the most “correct” realisation set, best describing the true spatial correlation.
